# Use of narsoplimab for eculizumab-refractory adult transplant-associated thrombotic microangiopathy (TA-TMA)

**DOI:** 10.1007/s00277-026-06756-0

**Published:** 2026-01-17

**Authors:** Sara Young, Indumathy Varadarajan

**Affiliations:** University of Virginia, 1215 Lee St, Charlottesville, VA 22903 USA

**Keywords:** Transplant associated, Thrombotic Microangiopathy (TA-TMA), Eculizumab, Narsoplimab, Renal failure, Diffuse alveolar hemorrhage

## Abstract

Transplant-associated thrombotic microangiopathy (TA-TMA) is a fatal complication associated with hematopoietic stem cell transplant (HSCT). Endothelial dysfunction and complement activation cause consumptive thrombocytopenia with intravascular hemolysis, resulting in end-organ damage, especially to the kidneys and lungs. There are no U.S. Food and Drug Administration (FDA)-approved agents for TA-TMA, although Eculizumab is the most commonly used agent to treat TA-TMA. Patients who do not respond to Eculizumab have a dismal prognosis, with reported mortality up to 80%. Narsoplimab, a mannan-binding lectin-associated serine protease-2 (MASP-2) inhibitor, has been shown to treat TA-TMA by inhibiting the lectin pathway of the complement cascade. We report the first adult case with successful management of eculizumab-refractory TA-TMA with Narsoplimab. Our patient received a matched unrelated donor (bone marrow) allogenic HSCT and subsequently developed multi-organ damage. He was refractory to numerous treatments, including eculizumab, steroids, rituximab, and plasma exchange. After developing diffuse Alveolar hemorrphage and renal failure, he was initiated on Narsoplimab and later achieved a complete hematological response and became transfusion independent. This case highlights the importance of early recognition of TA-TMA and the need to switch therapy to other complement inhibitors if resistance to Eculizumab is noted.

## Introduction

Transplant-associated thrombotic microangiopathy (TA-TMA) is a complication of hematopoietic stem cell transplantation (HSCT), resulting in significant mortality and morbidity [1]. TA-TMA has a median onset of 86 days post-allogeneic HSCT and occurs due to a combination of microvascular endothelial dysfunction and complement activation [2]. Insults sustained during stem cell transplant can lead to TA-TMA; these include conditioning chemotherapy, calcineurin inhibitors, infections, and graft versus host disease (GVHD), resulting in microangiopathic hemolytic anemia, thrombocytopenia, and thrombosis [2, 3]. The most common clinical manifestation of TA-TMA is renal failure. However, TA-TMA can also result in posterior reversible encephalopathy (PRES), diffuse alveolar hemorrhage (DAH), serositis, and gastrointestinal bleeding [4–6]. Currently, there is no FDA-approved agent for TA-TMA. Eculizumab, a C5 inhibitor, is usually the first line of treatment [5]. However, refractory TA-TMA has a dismal outcome, reporting > 80% mortality in pediatric patients [7]. Here, we report the first adult case of eculizumab-refractory TA-TMA responding to Narsoplimab, an inhibitor of mannan-binding lectin-associated serine protease-2 (MASP-2).

## Case presentation

A 24-year-old male received a 10/10 matched unrelated donor allogeneic HSCT marrow product for aplastic anemia. The recipient and donor were IgG positive for Epstein–Barr virus (EBV); however, the donor was negative for IgG cytomegalovirus (CMV), while the recipient had evidence of prior CMV exposure (CMV D-/R +, EBV D/R +). He received Fludarabine, Cyclophosphamide, antithymocyte globulin, and total body irradiation (2 Gy) for conditioning; GVHD prophylaxis with methotrexate starting on T + 1; and tacrolimus starting on T-3. He had a reactivation of CMV with a viral load of 517 IU/mL on T + 11; hence, he was treated with foscarnet during his admission for transplant. He was placed on letermovir prophylaxis once his viral load decreased to < 50 IU/mL. On T + 46, he developed fevers and diarrhea and was found to have an EBV reactivation with a viral load of 282,000 IU/mL (5.45 log). Colonoscopy with biopsies revealed a lymphoproliferative process. He was treated with four doses of weekly Rituximab with a resolution of the EBV viremia, and the post-treatment PET CT scan did not reveal any evidence of disease.

On T + 137, the patient was re-admitted for facial swelling and hypertension. He had progressive thrombocytopenia, hemolytic anemia with LDH of 1046 U/L, plasma-free hemoglobin of 170 mg/dL, haptoglobin < 8 mg/dL, and an absolute reticulocyte count of 0.15 m/uL with multiple schistocytes present in the peripheral smear. His urine protein to creatinine ratio was 6.7 mg/mg, with 24-h total urine protein of 4.85 g/dL. His C5b-9 complex level was 131 ng/mL (ref < 250 ng/ml). The patient was transitioned from sirolimus to mycophenolate for immunosuppression on T + 137. Weekly eculizumab at a dose of 900 mg was initiated within one week of hospitalization. Despite achieving therapeutic levels (eculizumab plasma level of 164 mcg/mL), the patient developed DAH, serositis, and worsening kidney function, leading to intubation on T + 155. Unfortunately, he further deteriorated and required dialysis on T + 166. By T+190 he had received 4 doses of 700 mg Rituximab, high-dose steroids 1 gm of Methy prednisilone for 3 days and Dexamethasone 10 mg Q6H for 1 week, and 20 episodes of approximately every other day therapeutic plasma exchange in addition to eculizumab. Despite combination therapy, there was no hematologic response, and he required daily platelet and RBC transfusions. His platelets continued to remain critically low < 10 × 10^^9^/L.

**Fig. 1:**
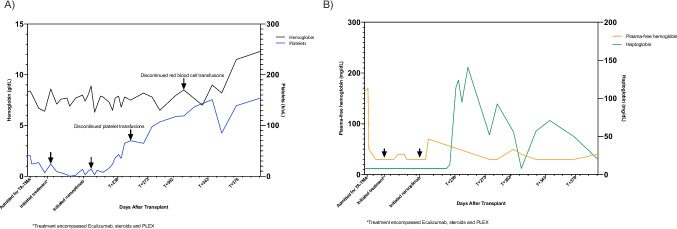
Trend in hemolytic parameters and CBC

Narsoplimab was obtained through a single-patient expanded access request from the manufacturer after submission of an Investigation New Drug application (IND) to the FDA, as it was not yet approved for TA-TMA. Narsoplimab at 370 mg was started on T + 197 and administered twice weekly. He had a detectable haptoglobin of 13 mg/dL on T + 232 and, as of T + 236, no longer required frequent platelet transfusions. After a prolonged complex hospitalization for 4.5 months with a 1-month stay in the intensive care unit, the patient was discharged to a rehabilitation center. The patient had received at least 75 units of packed red cells and greater than 100 units of platelets.The patient continued to respond to Narsoplimab treatment in the outpatient setting and became transfusion-independent on T + 302 (Fig. 1). He was given Narsoplimab until 17 months post-transplant, by which time he achieved a complete hematological response. At 25 months post-transplant, he is back home living a near-normal life with his family but continues to require renal replacement therapy.

## Discussion

TA-TMA has a long-standing challenge in diagnosis due to its spectrum of clinical presentations and lab abnormalities. The Jodele criteria and guidelines on diagnostic and prognostic assessment have helped with early identification and management based on risk stratification of TA-TMA [3]. Elevated C5b-9, random urine protein-to-creatinine ratio ≥ 1 mg/mg, organ dysfunction, LDH ≥ 2 times the upper limit of normal, concurrent grade II-IV GVHD, and concurrent infections are noted as poor prognostic factors [3]. Patients with any of these factors are classified as high-risk TA-TMA [3].

Eculizumab has demonstrated efficacy in pediatric patients with high-risk TA-TMA, improving overall survival (OS) to 71% at 6 months and 62% at one year compared to control (18% and 16.7%, respectively) [8]. Unfortunately, our patient was refractory to Eculizumab despite achieving therapeutic drug levels. Narsoplimab (Omeros Corporation, Seattle, USA) is a human immunoglobulin G4 monoclonal antibody that inhibits mannan-binding lectin-associated serine protease-2 (MASP-2), the effector enzyme of the lectin pathway of complement [9]. In a Phase II study, treatment with Narsoplimab showed promising results in patients with high-risk TA-TMA, with a 61% response rate (defined as improvement of TA-TMA markers and organ dysfunction) and 68% OS at 100 days from the date of diagnosis. However, this study did not include patients who were refractory to eculizumab [10]. Narsoplimab has shown some efficacy in infants and children having eculizumab-refractory TA-TMA [11]. A retrospective study looked at 5 pediatric patients, 1 infant, and 4 children, who presented with multi-organ dysfunction and GVHD. The study reported 1 complete response with resolution of sinusoidal obstruction syndrome. Two children had hematopoietic recovery but no organ recovery (partial response) [11].

This report demonstrates the effective use of Narsoplimab in treating an adult with TA-TMA, who had been refractory to multiple agents, including Eculizumab, rituxan, high-dose steroids, and therapeutic plasma exchange. Our case highlights and emphasizes the potential for treatment with Narsoplimab for refractory TA-TMA, which is otherwise fatal. High-risk TA-TMA can also occur without an elevated C5b-9 complex, especially with increased proteinuria. Narsoplimab has also been used successfully to treat high-risk TA-TMA while maintaining GVHD prophylaxis in the first-line setting [12]. Of note, Narsoplimab may be difficult to obtain in different countries and may require prolonged administration for optimal response, such as in our case. Additionally, our case highlights the importance of timely intervention, as injury induced to the glomeruli from TA-TMA can often be irreversible, and patients may require long-term renal replacement therapy. Narsoplimab was not able to reverse renal injury, and our patient is currently awaiting donor selection for a renal transplant. In a disease with high morbidity and mortality rate, Narsoplimab may be an effective therapy for patients with refractory TA-TMA.

## Competing interests

The authors declare no competing interests.

## Data Availability

No datasets were generated or analysed during the current study.
